# Buruli Ulcer in Liberia, 2012

**DOI:** 10.3201/eid2003.130708

**Published:** 2014-03

**Authors:** Karsor Kollie, Yaw Ampem Amoako, Julien Ake, Tarnue Mulbah, Fasseneh Zaizay, Mohammed Abass, Linda Lehman, Albert Paintsil, Fred Sarfo, Clement Lugala, Alexandre Tiendrebeogo, Richard Phillips, Kingsley Asiedu

**Affiliations:** Neglected Tropical Diseases Control Program, Monrovia, Liberia (K. Kollie, T. Mulbah);; Komfo Anokye Teaching Hospital, Kumasi, Ghana (Y.A. Amoako, F. Sarfo, R. Phillips);; Medical Assistance Program International West Africa Region, Abidjan, Côte d’Ivoire (J. Ake, F. Zaizay);; Agogo Presbyterian Hospital, Agogo, Ghana (M. Abass);; American Leprosy Missions, Greenville, South Carolina, USA (L. Lehman);; Korle Bu Teaching Hospital, Accra, Ghana (A. Paintsil);; World Health Organization (WHO), Monrovia (C. Lugala);; WHO, Brazzaville, Republic of Congo (A. Tiendrebeogo);; WHO, Geneva, Switzerland (K. Asiedu)

**Keywords:** Buruli ulcer, Liberia, assessment, control, bacteria, Mycobacterium ulcerans, tuberculosis and other mycobacteria

**To the Editor:** Buruli ulcer, a necrotizing skin disease caused by *Mycobacterium ulcerans*, is highly endemic to West Africa (*1*,*2*) and is characterized by large ulcerations on the lower limbs (60% of cases) as well as on the upper limbs (30%) and other parts of the body (10%). Although the mode of transmission is unknown, most cases of Buruli ulcer occur around swampy and riverine areas; children <15 years of age are most often affected (*2*,*3*). The recommended treatment consists of a combination of daily oral rifampin and intramuscular streptomycin for 8 weeks, supplemented by wound care when appropriate (*4*). Large ulcers may require debridement and grafting to facilitate wound closure, and physiotherapy is often indicated to prevent functional limitation, particularly for lesions located over joints.

In Liberia, 2 Buruli ulcer patients were reported in 1981 in the Foya region, along the Manor River basin; 4 more patients were observed in the same area in 1984 (*5*). Since then, some patients from Liberia have received treatment for Buruli ulcer in Côte d’Ivoire (*6*), and suspicious cases have been detected in some parts of Liberia since the end of a civil war in 2004. 

Recently, Buruli ulcer cases have been suspected in 3 counties of Liberia, Bong, Lofa, and Nimba; these regions share borders with the Buruli ulcer–endemic regions of Côte d’Ivoire and Guinea. During 2012, the government of Liberia, with assistance from the Medical Assistance Program International and with technical support from the World Health Organization (WHO), conducted a rapid status assessment in these 3 counties. In January 2012, a core team of national and county health personnel was trained in the recognition and assessment of Buruli ulcer. Assessment was conducted during February 18–27, 2012, by a team made up of those who had received the preassessment training and WHO Buruli ulcer consultants.

During the preassessment training, notice was given to all health facilities to record all lesions with features suggestive of Buruli ulcer. The persons identified during this period then came to the nearest health facility to be examined by the assessment team or were traced to their homes. A detailed history was collected and physical examination conducted, and swab specimens and fine-needle aspirates (2 for each lesion) were obtained for confirmation of diagnosis by classical PCR (*7*).

On the basis of the WHO case definition for Buruli ulcer (*1*), 60 of 181 persons screened were suspected to have Buruli ulcer. All cases were documented by photography and registration on a modified version of the WHO Buruli ulcer assessment form (*1*). For 21 (35%) of the 60 patients, *IS2404* PCR testing at Komfo Anokye Teaching Hospital in Ghana confirmed the clinical diagnosis; these patients received the recommended treatment for Buruli ulcer. Those with negative test results received wound care and supportive management. 

A total of 21 confirmed cases occurred: 9 in Nimba County, and 6 each in Bong and Lofa Counties (Figure). Nine (35%) of the 21 patients were children <15 years of age; 11 patients were male and 10 female. Most (17 of 21) lesions were on the lower limbs; 3 were on the upper limbs and 1 on the thorax. Fifteen patients had ulcers, 2 edema, and 3 osteomyelitis. No lesions were classified as category I, but 11 (52.4%) were category III.

**Figure:**
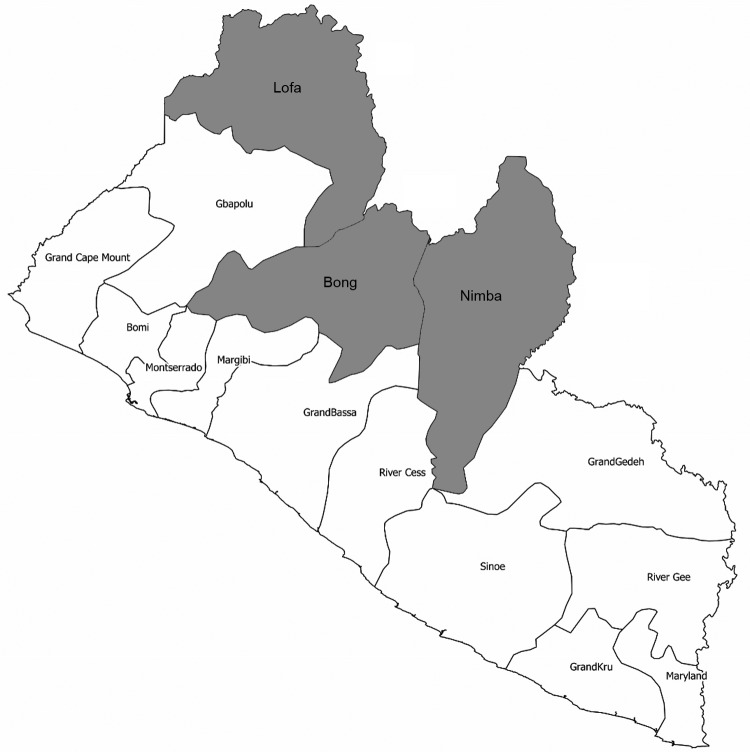
Counties in which cases of Buruli ulcer were found during 2012 (gray shading), Liberia.

Our findings suggest that Buruli ulcer in Liberia may be more prevalent than previously thought. Although only 3 of 15 counties were assessed, results show that Buruli ulcer has not disappeared from Liberia and that the absence of regular reporting should be investigated. A long civil war and lack of familiarity with the disease by health care workers may have contributed to poor reporting.

It has been almost 3 decades since the last published report of Buruli ulcer cases in Liberia (*5*), but other studies have found the disease in countries many years after it was last reported. In Cameroon, a case search in 2001 in 2 districts where cases had last been reported 24 years earlier found 436 active and inactive cases (*8*). In southwestern Nigeria, a case search in 2006 found 14 active and inactive cases 30 years after the most recent publication (*9*). More recently, in 2012, a similar situation was reported in Gabon (*10*).

Several measures might improve Buruli ulcer control and surveillance in Liberia. First, treatment and control activities should be included in the Neglected Tropical Diseases Control Program at all levels to enhance surveillance. Second, health workers at all levels should be trained to recognize the disease. Third, a detailed assessment of the extent of Buruli ulcer in the 3 counties visited as well as in other counties should be prepared. Fourth, partner/donor support for Buruli ulcer activities should be enhanced. Fifth, capacity of the National Reference Laboratory to be able to perform PCR for confirmation of Buruli ulcer cases should be expanded. Last, Buruli ulcer should be incorporated into the national surveillance system to enable better data collection.
